# Knowledge, Practice and Self-Reported Confidence Level of Croatian Dentists in the Use of Local Anesthesia: A Cross-Sectional Study

**DOI:** 10.3390/healthcare11142006

**Published:** 2023-07-12

**Authors:** Antonija Tadin, Klaudia Aleric, Daniel Jerkovic, Lidia Gavic

**Affiliations:** 1 Department of Restorative Dental Medicine and Endodontics, Study of Dental Medicine, University of Split School of Medicine, 21000 Split, Croatia; klaudia.aleric13@gmail.com (K.A.); lgavic@mefst.hr (L.G.); 2 Department of Oral and Maxillofacial Surgery, Clinical Hospital Centre Split, 21000 Split, Croatia; daniel.jerkovic@mefst.hr

**Keywords:** local anesthesia, knowledge, dentists, self-confidence, adverse effects

## Abstract

**Objectives**: To provide safe and effective local anesthesia, dentists must have knowledge of neuroanatomy, anesthesia agents, techniques, equipment, and proper use of local anesthetics. This study aims to explore the knowledge, practices, and confidence regarding local anesthetics and anesthetic techniques in dentistry. **Material and Methods**: The online cross-sectional questionnaire was conducted via social media, and yielded 441 responses from across the country (69.8% women and 30.2% men; 70.7% general dentists; and 29.3 specialists). The data collected included sociodemographic characteristics, knowledge, and practices pertaining to the usage of local anesthesia. The questionnaire also documents their self-assessed confidence level in applying different local anesthetic techniques and experiences with adverse reactions. The obtained data were processed by description and using a generalized linear model for regression. **Results**: The respondents had a median knowledge score of 6 out of a possible 14 points regarding local anesthetics in dental medicine, and their median self-confidence level in the successful application of various techniques of local anesthesia was 54 out of a maximum of 85 points. The results showed that a higher knowledge level was associated with the female gender (OR 1.83, CI 1.13–2.98, *p* = 0.014) and specialization in oral surgery (OR 7.04, CI 1.71–29.07, *p* = 0.007). In contrast, a lack of confidence in using various local anesthetic techniques was also associated with the female gender (OR 0.63, CI 0.41–0.99, *p* = 0.047) and specialization in orthodontics (OR 0.16, CI 0.03–0.88, *p* = 0.035). Of the respondents, 81.4% (n = 371) experienced a local complication, and 42.2% (n = 186) experienced a systemic complication during local anesthesia. The complications experienced cannot be associated with a lack of knowledge or self-confidence (*p* > 0.05). The majority of respondents (364 of the 441 total—82.5%) expressed interest in receiving further education on the topic of local anesthesia. **Conclusions**: The research results show that the dentists involved in the study have poor knowledge of local anesthetics and moderate self-reported confidence levels in using various local anesthetic techniques. Moreover, dentists’ self-confidence in applying different techniques of local anesthesia is not related to their knowledge. Therefore, it would be necessary for dentists to undergo a continuing dental education program that enables them to enhance their skills and knowledge in local anesthesia.

## 1. Introduction

Local anesthesia is an indispensable part of clinical practice in dental procedures, used for intraoperative pain management, diagnostics, and therapeutic purposes. Its administration is crucial in preventing pain and anxiety during dental procedures, promoting a positive attitude towards the practitioner, and facilitating treatment [1,2].

Local anesthesia in dentistry refers to the temporary loss of sensation, including pain, in a specific part of the body. This effect is achieved by applying or injecting a locally acting agent. Several types of local anesthetics and anesthesia techniques are available in dental medicine, each with its own advantages and disadvantages. Dentists must consider multiple factors when choosing a local anesthesia agent and a technique for a particular procedure. These include the location and extent of the area being treated, the patient’s medical history and any allergies or sensitivities they may have, the procedure being performed, and the expected duration [1,2].

One of the advantages of local anesthesia in dentistry is that it is a relatively safe and uncomplicated procedure. However, local anesthesia also has some disadvantages and may not be suitable for all patients. In some cases, administering anesthesia can be risky. Reactions to local anesthetics can be various, and there is always the possibility of failure during the procedure. Dentists should be aware of these potential occurrences when using a dental anesthetic. Furthermore, there is a possibility of interaction between anesthetics and other drugs, which range from mild and tolerable to severe and dangerous, whether local or systemic, or temporary or permanent. To perform safe and successful anesthesia, dentists must understand the types of anesthesia and their applications, patient assessment and preparation, pharmacology and drug interactions, and possible complications and their treatment [2,3,4,5,6,7,8,9,10]. By taking these considerations into account, it is crucial for dental practitioners to continuously improve their knowledge, competence, and practice, including their understanding of side effects, contraindications, and interactions, to provide optimal treatment for each patient [11].

After reviewing the existing literature, it becomes apparent that the conducted studies have focused only on reassessing dentists’ knowledge of the maximum dosage and dosage calculation of local anesthetics. The results consistently indicate that dosage problems persist because dentists commonly depend on the number of ampoules rather than adhering to recommended dosages [12,13,14,15]. Moreover, various studies have examined the incidence of systemic or local complications arising from the administration of local anesthetics in dentistry, alongside appropriate management strategies [15,16,17,18]. It is crucial to acknowledge that the safety and confidence levels associated with administering anesthesia injections have solely been evaluated among dental students, not professional dentists [19,20,21]. Based on the information provided, it can be inferred that there is a limited availability of literature concerning knowledge, practice, and confidence in administering local anesthetics among dental practitioners in Croatia and worldwide. Thus, the objectives of this study were to assess (1) knowledge and practice regarding local anesthetics, (2) self-confidence level in its administration, and (3) experience of local and systemic complications associated with applying local anesthetics in dental practice. To the best of our knowledge, this is the first study in Croatia that associates the knowledge and confidence in managing local anesthetics with the incidence of adverse events and reactions in the dental office.

## 2. Materials and Methods

### 2.1. Study Design and Population

A national observational, cross-sectional study was conducted at the Department of Restorative Dental Medicine and Endodontic School of Medicine at the University of Split in Croatia in January 2023. Data were collected using the Google survey tool (Google Forms), and the survey link was disseminated through social media sites. Participants were requested to share the survey link with their connections after completing it. The participation was voluntary and anonymous, and participants were informed at the beginning of the survey that their participation was considered consent. Manuscript methods were carried out following relevant guidelines and regulations (Declaration of Helsinki and Strengthening the Reporting of Observational Studies in Epidemiology) [22]. The School of Medicine Ethics Committee approved our study protocol (Class: 003-08/22-03/0003, No: 2181-198-03-04-22-0081).

A total of 441 dentists participated in the study. The median age was 35 years (IQR 29–44, min 25, max 65), and the majority were women (69.8%) (Table 1). The study targeted dental practitioners working in Croatia who were capable and willing to respond to an online survey and had at least one year of clinical experience. The exclusion criteria were as follows: incompletely filled questionnaires and dental practitioners who were retired or not actively engaged in clinical practice (i.e., working outside dental medicine). Respondents were selected using a convenience sampling method that combined purposive and snowball sampling.

The minimum required sample size (n = 351) was calculated using Sample Size Calculator (Inc.RaoSoft^®^, Seattle, WA, USA), an online sample size calculator, based on an estimated population of 3928 dental practitioners employed in the Croatian health care system (511 specialists and 3417 general dentists), an anticipated response rate of 50%, a confidence level of 95%, and a 5% margin of error [23].

### 2.2. Questionnaire

The questionnaire was developed and adapted from similar studies on local anesthetics in dental practice [12,13,14,24,25,26,27]. The questions were reviewed and evaluated by a working group consisting of three dental practitioners who are university professors specializing in endodontics, pedodontics, and oral surgery. They assessed the content validity of the questionnaire. The survey was then pilot tested on a sample of 15 dental practitioners to ensure the survey’s transparency, acceptability, and readability. In addition, the pilot study estimated the time required to complete the questionnaire to be around 15 minutes. The subjects of the pilot survey were not included in the primary data. The pilot study utilized Cronbach’s alpha coefficient test to assess internal consistency. The findings revealed that Cronbach’s alpha for the knowledge scores, consisting of 7 items, was 0.712, while the internal consistency for the confidence score in the application of different local anesthesia techniques, comprising 17 items, yielded an alpha value of 0.698. These results indicate acceptable reliability [28].

The self-administered questionnaire consisted of 50 questions divided into five sections. The first section of the questionnaire contained seven general data questions about the dental practitioners, including gender, age, education level, specialization, practice setting, years in dental practice, and the number of patients per working day. The second section of the questionnaire consisted of seven multiple-choice questions meant to assess the knowledge about local anesthesia in dental practice. The knowledge-related questions were formulated based on the recommendations provided in Malamed’s Handbook of Local Anesthesia [10]. There were five questions with one correct answer and two with multiple correct answers, which were evaluated with a partial credit scoring method [29]. The participants’ overall knowledge of local anesthesia in dentistry was evaluated by calculating the sum of their correct answers, with a maximum possible score of 14. The third section of the questionnaire contained 10 items related to the dental practitioners’ experience regarding local anesthesia and self-assessment of knowledge, education, and practice. In the fourth section, dental practitioners were presented with 17 questions where they were required to rate their confidence level in their ability to administer various local anesthesia techniques. This assessment was conducted using a five-level Likert scale (1—not confident, 2—slightly confident, 3—somewhat confident, 4—fairly confident, and 5—very confident). The total score was calculated by summing up the points obtained on each answer, while the maximum score of 85 points represented the most positive confidence level. According to Bloom’s cutoff order, respondents’ overall knowledge and self-confidence were considered good when the score ranged between 80% and 100%, moderate when the score fell between 60% and 79%, and low when the score was below 60% [30]. In the final fifth section, dentists were provided with eight questions related to the occurrence of adverse effects during the administration of local dental anesthesia. These questions aimed to gather information on any experienced local or systemic adverse reactions during their clinical work with patients. Additionally, dentists were asked about the specific anesthetic agent and technique of local anesthesia used when the complication occurred. Furthermore, one question focused on determining the most frequently utilized anesthetic agent in dental practice.

#### Data Analysis

SPSS Statistics version 26.0 (IBM Corp., Armonk, NY, USA) was used for data analysis, and results were interpreted at a significance level of *p* < 0.05. Data normality was tested using the Kolmogorov–Smirnov test. Descriptive analysis was utilized and expressed as frequency and percentage for categorical variables. Due to the non-normal distribution of the data, continuous variables were presented as the median (interquartile range, IQR), while categorical variables were presented with frequencies and percentages. Generalized linear model (GLM) analysis was used to identify the characteristics associated with knowledge and confidence level scores, including gender, age in years, education level, specialization, years in dental practice, number of patients per working day, and experienced local or systemic complication as independent variables.

## 3. Results

Table 1 shows the sociodemographic data of dental practitioners. The study was conducted with 441 dentists, of whom 69.8% were women and 30.2% were men. Among them, 312 were general dentists, and 129 were specialists in different areas of dentistry.

Table 2 shows the frequency of correct and incorrect answers to the questions about local anesthetics. The median of scores of correct answers in the objective knowledge test of all dentists was 6, with an interquartile range of 4 to 8 (min 0, max 12). A low level of knowledge was shown by 40.8% of respondents, whose knowledge was below the median. Dentists showed the best result when asked about the advantages of administering a local anesthetic combined with a vasoconstrictor. Almost all respondents knew that the vasoconstrictor reduces bleeding (97.7%), 83.2% knew that it improves the anesthetic onset and duration, and 69.8% knew that it decreases the systemic absorption rate of local anesthetics.

Table 3 presents the results of dental practitioners’ self-reported knowledge and experience regarding local anesthesia. The majority of respondents (74.8%) self-assessed their knowledge about local anesthesia as average. Among the dentists, 72.8% reported that they always check the patient’s medical history before administering local anesthesia. Additionally, 29.0% of the respondents stated that they always aspirate before administering a local anesthetic, while 62.2% reported aspirating only in block techniques.

Table 4 shows the self-confidence level among dental practitioners during the administration of 17 different dental anesthesia techniques. Dentists expressed the highest level of self-confidence when administering supraperiosteal injections (78.2%), followed by the periodontal ligament technique (47.8%) and the inferior alveolar nerve block (40.6%). Conversely, dentists reported the least confidence when applying the sphenopalatine nerve block (5.2%) and the Vazirani–Akinosi technique (7.5%). The median self-assessed confidence level for all dentists in applying various local anesthesia techniques in dentistry was 54, with an interquartile range of 41 to 63 (minimum 17, maximum 85). Nearly half of the respondents (49.9%) showed a low level of self-confidence in applying dental anesthesia techniques.

Table 5 presents the experienced adverse reactions to dental local anesthetics reported by the respondents. Among the respondents, 84.1% reported experiencing local complications associated with dental local anesthetics, while 42.2% reported experiencing systemic reactions. Syncope was the most common systemic reaction, reported by 37.6% of the respondents. The most frequent adverse reactions were observed with the use of articaine, which happened to be the most commonly utilized anesthetic among the dentists in the study. Specifically, 56.7% of the dentists used 4% articaine HCl with epinephrine 1:100,000, while 26.5% used 4% articaine HCl with epinephrine 1:200,000. In addition to articaine, other frequently used anesthetics among the dentists in the study included lidocaine with epinephrine 1:100,000 (5.2%), lidocaine HCl 2% with epinephrine 1:50,000 (4.5%), lidocaine HCl 2% plain (1.6%), and 3% mepivacaine HCl plain (3.9%).

After adjustments of dentists’ characteristics, higher theoretical knowledge level was associated with the female gender (OR 1.83, CI 1.13–2.98, *p* = 0.014) and specialization in oral surgery (OR 7.04, CI 1.71–29.07, *p* = 0.007). Conversely, lower knowledge is associated with older age and more patients per day. Lack of self-confidence when applying different techniques of local anesthesia in dentistry can be linked to the female gender (OR 0.63, CI 0.41–0.99, *p* = 0.047) and specialization in orthodontics (OR 0.16, CI 0.03–0.88, *p* = 0.035). Oral surgeons showed the highest level of confidence compared to general dentists (OR 4.91, CI 1.29–18.72, *p* = 0.020) (Table 6).

The correlation between the assessment of dental practitioners’ knowledge and the overall score of self-assessed confidence is positive (*r* = 0.72) and not statistically significant (*p* = 0.129, Spearman’s *ρ*).

## 4. Discussion

The aim of this study was to assess dentists’ knowledge and practices pertaining to local anesthetics, local anesthetic techniques, and associated adverse effects. The goal was to establish a comprehensive foundation that can be utilized to enhance their knowledge and attitude toward this subject. Upon reviewing the existing literature, it was found that only a limited number of studies have investigated dentists’ understanding of local anesthetics [5,12,13,14,24,31], and a few more studies have evaluated complications associated with their usage and subsequent treatment [2,8,9,32,33]. To date, no study has been conducted on this topic in Croatia. Therefore, there is a significant gap in the available information concerning this subject, particularly regarding the relationship between knowledge of local anesthetics in dentistry, confidence in utilizing various techniques, and the occurrence of complications.

Providing adequate dental local anesthesia is a valuable and fundamental clinical skill for oral health professionals. The results of this study show that most dentists need to learn the correct dosage of each anesthetic, the maximum amount of vasoconstrictor that can be used, and the contraindications of its usage. The dentists surveyed showed low knowledge of local anesthetics, with a median score of 6 out of a maximum of 14. Only 43.1% of the respondents knew the maximum dose of lidocaine with a vasoconstrictor, while the results of the study conducted in Saudi Arabia were even unsatisfactory and insufficient. There, only 31% of respondents gave the correct answer to the question about the maximum dose of lidocaine with a vasoconstrictor, and only 15% of respondents knew the maximum number of ampoules of lidocaine for a healthy adult [14]. In a study conducted among academic deans of various dental schools in the USA, it was found that there is inconsistency in teaching practices regarding the maximal safe doses for local anesthesia in the U.S. Different schools rely on two different dosage standards mentioned in textbooks, such as those by Malamed and Goodman and Gilman, as well as guidelines published by the Council on Dental Therapeutics and the American Academy of Pediatric Dentistry [10,13]. It is possible that the various learning approaches of the undergraduate studies attended by the participants of this study contributed to these unfavorable results.

According to the research findings, a significant proportion of respondents exhibited inadequate practices regarding aspiration during anesthetic administration. Specifically, 4.8% of respondents reported never aspirating, while 29% consistently performed aspiration during all techniques of local anesthesia application. Additionally, 66.2% of respondents indicated aspirating only during block anesthesia. A similar study conducted in Saudi Arabia revealed even higher rates of neglecting aspiration, with 53% of respondents reporting never aspirating during anesthetic administration. Among those who did aspirate, 43% only did so when using a lower alveolar block, while a mere 4% consistently performed aspiration across all local anesthesia techniques [14]. It is worth noting that some authors have recommended a minimum of two negative aspirations before anesthetic administration, highlighting the importance of this practice. However, it is evident that many dentists frequently overlook the significance of aspiration and the potential adverse consequences associated with intravascular administration of anesthetics [34,35,36].

Confidence is a crucial attribute that encompasses the ability to successfully carry out procedures without fear of failure, along with a strong belief in one’s own skills and abilities. This essential quality significantly influences clinical performance, interactions with patients, and overall job satisfaction among healthcare professionals [37,38]. In this study, the dentists who responded demonstrated a moderate level of self-confidence when applying various techniques of local anesthesia, as indicated by a median score of 54 out of a maximum possible score of 85. Lower self-confidence in using various local anesthetic techniques was associated with the female gender (*p* = 0.047) and specialization in orthodontics (*p* = 0.035). In contrast, higher self-confidence was associated with specialization in oral surgery (*p* = 0.020). Studies on dentists and dental students have consistently confirmed that men exhibit higher levels of self-confidence in various dental clinical procedures. This increased self-confidence in men can be attributed to their lower fear of performing these procedures and their higher experience in performing a greater number of dental procedures [38,39,40]. Interestingly, the findings of this study revealed that although men exhibited greater confidence in utilizing various local anesthetic techniques, they did not demonstrate superior knowledge in this area (*p* = 0.014). It is already well known that self-assessment of confidence is not necessarily correlated with higher levels of knowledge and skills. This phenomenon can be attributed to the Dunning–Kruger effect, which explains how individuals with inflated self-perceived confidence may actually possess lower levels of competence [41,42,43]. Additionally, studies conducted among dental students have consistently shown that higher self-confidence tends to be associated with lower academic performance, while lower self-confidence is observed among higher-performing students [37,44].

Dentists who participated in surveys indicated that they had the highest level of confidence in utilizing supraperiosteal anesthesia (78.2%), the periodontal ligament technique (47.8%), and the inferior alveolar nerve block (40.6%). On the other hand, they expressed the least confidence in performing the sphenopalatine nerve block (5.2%), the Vazirani–Akinosi technique (7.5%), and Gow-Gates mandibular block (11.3%). According to the study from Pakistan conducted on dentists with and without specialization, 51.3% of the respondents reported that they knew the technique for applying the Gow-Gates mandibular block, but their self-assessment of competence in successfully performing this technique was only 5.14 on a scale of 0 to 10 [45]. Based on the provided information, it can be inferred that dentists exhibit higher confidence levels and a greater frequency of usage when employing the lower alveolar block technique compared to the Gow-Gates mandibular block technique. This suggests that dentists may feel more comfortable and familiar with the lower alveolar block technique, leading to its preferred use in clinical practice.

Previous studies conducted with students and dentists have consistently indicated that they feel inadequately prepared for the utilization of local anesthesia in general practice. They often express a lack of confidence in their knowledge of the anatomical aspects of different techniques and their ability to manage complications associated with local anesthesia administration [46,47]. The reported failure rates in applying local anesthesia techniques range from 15% to 30%. Various factors have been associated with this issue, including anatomical, biochemical and physiological, pathological, psychological, and operative factors. Additionally, challenges related to the anesthetic solution itself and the use of a specific armamentarium can contribute to the complexity of the issue [48,49].

Local and systemic side effects were observed in a significant proportion of the respondents in our study, with 84.1% experiencing local effects and 42.2% experiencing systemic effects. These adverse reactions were most frequently associated with the use of articaine, which also happened to be the most commonly utilized anesthetic among the dentists surveyed. Specifically, 56.7% of dentists employed 4% articaine HCL with epinephrine 1:100,000, while 26.5% used 4% articaine HCL with epinephrine 1:200,000. Similar findings were reported in a study conducted in Germany [5]. Articaine is widely used in various European and worldwide countries, including Germany, Austria, Italy, France, Bulgaria, the Netherlands, and India [5,50,51], while in the United States and the United Kingdom, lidocaine emerged as the most commonly utilized local anesthetic in dental practice, followed by articaine [5]. Plain mepivacaine usage was reported by 3.9% of respondents in this study, aligning with its shorter duration of action and specific indications for use. This pattern of usage is consistent with trends observed in other countries across the globe [3,4,5,6].

Medical emergencies were most likely to occur during and after the application of local anesthesia. Psychomotor reactions (37.6%) and injection site complications (51.7%) were the most frequently reported adverse effects. The findings of this study align with previous studies, where syncope was identified as the most common complication [8,52,53]. Furthermore, systemic and local complications were most prevalent when employing the inferior alveolar nerve block technique and buccal infiltration, corroborating findings from other studies [2]. Notably, articaine has been linked to an increased risk of paresthesia following inferior alveolar nerve block, with 14.1% of dentists reporting paresthesia as a complication in their patients [54]. Among the systemic complications reported by participants in this study, a significant number of allergic reactions and anaphylaxis (3.2%) were listed. However, the literature indicates that allergic reactions to local anesthetics are rare, and most adverse effects are psychogenic/vasovagal. Dentists sometimes misinterpret vague clinical symptoms as allergic reactions and never ask for diagnostic confirmation. Therefore, it is possible that in our study, some participants associated milder reactions with more severe complications such as allergic reactions or anaphylaxis [9,33,55].

A comprehensive evaluation of a patient’s medical condition is a fundamental aspect of dental care provision. It involves meticulous documentation of the patient’s medical history and accurate assessment of baseline vital signs. These steps are crucial for dentists to effectively determine the patient’s suitability for anesthesia and the planned treatment. By thoroughly assessing the patient’s medical status, dentists can ensure the safety and well-being of their patients throughout the dental procedure [56]. Over 70% of the respondents in this study consistently perform medical anamnesis checks, reviewing the patient’s medical history, current medications, and potential allergies to anesthetics prior to administering local anesthesia. This process of history checking typically requires approximately two to five minutes. Taking a thorough medical history using appropriate tools has been proven to help dental students enhance their confidence in dealing with issues related to at-risk patients, as well as improve their knowledge of risk classification [57]. It is crucial to highlight the significance of obtaining a comprehensive medical history regarding previous reactions to local anesthetics. This step is essential to prevent misclassifying allergic reactions that have not been confirmed through appropriate testing. By diligently documenting prior reactions, healthcare providers can avoid unnecessary avoidance of local anesthetics or unnecessary delays in performing necessary surgical procedures that rely on their utilization. This approach ensures that patients receive the appropriate anesthetic while minimizing the potential for adverse reactions and optimizing the efficiency of dental interventions [58].

This study has several limitations that need to be acknowledged. Firstly, it relied on an online cross-sectional survey, meaning the data collected were based on self-reported knowledge and practices. The study’s sample was also limited to dentists active on social media platforms and networking applications. This could lead to a selection bias, as those who are more active or have a more substantial interest in the research topic may be more likely to participate. It is also possible that individuals who needed to learn more about the study topic may have chosen not to participate, which could impact the generalizability of the findings. Another limitation is the potential presence of the social desirability bias, where participants may respond in a manner they perceive as more socially acceptable or favorable [59]. This bias could impact the validity of the results and distort the actual prevalence or behavior being studied. The study also demonstrated a potential selection bias regarding the number of participating specialists. The proportion of specialists in the study sample was higher than the overall proportion of specialists among dentists in Croatia [23]. Lastly, the gender distribution of the study participants was skewed, with more women participating than men. It is common in online studies due to women’s higher engagement in social networks and willingness to complete surveys [60]. Considering these limitations, it is important to interpret the study findings cautiously and recognize the potential biases that may have influenced the results.

Local anesthesia is considered essential in modern medicine because it relieves pain, increases patient comfort, and leads to better outcomes. However, ignorance of proper use can have serious consequences, affecting patient comfort and undermining the credibility of medical personnel. This can be improved through comprehensive education, standardized guidelines, ongoing professional development, interdisciplinary collaboration, and quality assurance within the medical community [3,8,11,35,51,53]. Although dentists in Croatia must regularly attend dental medicine courses and congresses to renew their work license, governing bodies, such as the dental chamber responsible for their education, should make a more significant effort to design lectures and workshops on the topics of local anesthesia, as well as the prevention and therapy of complications that may arise during its application.

## 5. Conclusions

The study results revealed a lack of knowledge and self-confidence among dentists regarding the administration of local anesthesia. Additionally, the findings suggest a potential correlation between the gender of the therapist, specialization in oral surgery, and their level of knowledge and self-confidence in local anesthesia. The study found that better knowledge about local anesthetics was associated with the female gender, specialization in oral surgery, and younger age of the respondents. On the other hand, greater self-confidence in applying different techniques was related to the male gender and specialization in oral surgery. However, it is essential to note that the results of the study also indicate that knowledge alone does not necessarily correspond to confidence in utilizing different local anesthesia techniques.

## Figures and Tables

**Table 1:** Demographic and professional characteristics of the dental practitioner.

Characteristic		Frequency (n)	Percentage (%)
Gender	Male	133	30.2
	Female	308	69.8
Age—group (years)	≤30	156	35.4
	31–40	128	29.0
	41–50	105	23.8
	≥50	52	11.8
Academic qualification	DMD	359	81.4
	Master of Science	21	4.8
	Doctor of Philosophy	61	13.8
Specializations in dentistry	General dentist (not specialists)	312	70.7
	Endodontics	30	6.8
	Oral surgery	23	5.2
	Oral medicine	4	0.9
	Pediatric dentistry	18	4.1
	Orthodontics	10	2.3
	Periodontics	15	3.4
	Prosthodontics	21	4.8
	Family dentistry	8	1.8
Practice setting	Private practice—primary care	233	52.8
	Health center—primary care	121	27.4
	Secondary and tertiary care	66	15.0
	Dental study	21	4.8
Number of patients per day	1–5	30	6.8
	6–10	214	48.5
	≥11	197	44.7
Working experience (years)	1–5	167	37.9
	6–10	76	17.2
	11–20	111	25.2
	≤21	87	19.7

Data are presented as numbers and percentages.

**Table 2:** The frequency distribution (%) of dental practitioners’ answers to the questions regarding local anesthetics’ use in dental practice—knowledge test.

Question		Frequency (n)	Percentage (%)
The advantages of administering an LA combined with a vasoconstrictor (multiple-choice question)	*Improves the anesthetic onset and duration*	367	83.2
	*Reduces bleeding*	431	97.7
	*Decreases the systemic absorption rate of local anesthetics*	308	69.8
	I do not know	1	0.2
Contraindications to vasoconstrictors in dental practice (multiple-choice question)	*Heart diseases (unstable angina, recent myocardial infarction, recent coronary artery bypass surgery, untreated or uncontrolled congestive heart failure…)*	379	85.9
	*Uncontrolled hyperthyroidism*	18	4.1
	*Uncontrolled diabetes*	156	35.4
	*Sulphite allergies*	110	24.9
	*Steroid-dependent asthma*	10	2.3
	*Pheochromocytoma*	219	49.7
	I do not know	20	4.5
For normal healthy adults, the individual maximum FDA recommended dose of 2% lidocaine HCl with epinephrine	*7 mg/kg*	139	43.1
	6.6 mg/kg	112	25.4
	I do not know	190	43.1
For normal healthy adults, the individual maximum FDA recommended dose of 4% articaine HCl with epinephrine	*7 mg/kg*	150	34.0
	6.6 mg/kg	101	22.9
	I do not know	190	43.1
For normal healthy adults, the individual maximum FDA recommended dose of 3% mepivacaine HCl without epinephrine	7 mg/kg	52	11.8
	*6.6 mg/kg*	113	25.6
	I do not know	276	62.6
For patients with clinically significant cardiovascular impairment (ASA III or IV) the maximum recommended dose of epinephrine per appointment	0.2 mg (200 µg)	28	6.3
	*0.04 mg (40 µg)*	176	39.9
	I do not know	237	53.7
For normal, healthy patient (ASA I) the maximum recommended dose of epinephrine per appointment	*0.2 mg (200 µg)*	204	46.3
	0.04 mg (40 µg)	28	6.3
	I do not know	209	47.4

Data are presented as numbers and percentages. Correct answers are italicized.

**Table 3:** Dental practitioners’ self-reported knowledge and experience regarding local anesthesia.

Characteristic		Frequency (n)	Percentage (%)
Self-assessment of personal knowledge about local anesthesia in dental practice	Very bad	1	0.2
	Bad	13	2.9
	Average	330	74.8
	Good	83	18.8
	Excellent	14	3.2
Perceived sufficient level of education on local anesthesia during dental graduate and postgraduate studies	Yes	211	47.8
	No	148	33.6
	I do not know	82	18.6
Interested in further education on the topic of local anesthesia	Yes	364	82.5
	No	46	10.4
	I do not know	31	7.0
Checking for the medical history of patients prior to administration of local anesthesia	Always	321	72.8
	Very often	68	15.4
	Sometimes	39	8.8
	Rarely	11	2.5
	Never	2	0.5
Checking for medication usage prior to administration of local anesthesia	Always	324	73.5
	Very often	68	15.4
	Sometimes	34	7.7
	Rarely	14	3.2
	Never	1	0.2
Checking for the use of recreational drugs prior to administration of local anesthesia	Always	126	28.6
	Very often	50	11.3
	Sometimes	77	17.4
	Rarely	103	23.4
	Never	85	19.3
Checking for patient allergies prior to administration of local anesthesia	Always	320	72.6
	Very often	47	10.7
	Sometimes	41	9.3
	Rarely	17	3.9
	Never	16	3.6
The average time spent checking a medical history	I don’t check it	8	1.8
	≤1 min	134	30.4
	2–5 min	266	60.3
	≥5 min	33	7.5
The average number of local anesthetics administered per working day	˂3	87	19.7
	3–5	145	32.9
	>5	209	47.4
Performing aspiration prior to the administration of local anesthesia	Never	21	4.8
	In all techniques	128	29.0
	Only in block techniques	292	66.2

Data are presented as numbers and percentages.

**Table 4:** Self-confidence level among dental practitioners during administration of different dental anesthesia techniques.

Dental Anesthesia Techniques	Self-Confidence Level				
	Very Confident n (%)	Fairly Confident n (%)	Somewhat Confident n (%)	Sightly Confident n (%)	Not Confident n (%)
Supraperiosteal injections	345 (78.2%)	55 (12.5%)	35 (7.9%)	2 (0.5%)	4 (0.9%)
Middle-superior alveolar nerve block	85 (19.3%)	82 (18.6%)	112 (25.4%)	61 (13.8%)	101 (22.9%)
Middle and anterior-superior alveolar nerve block	78 (17.7%)	76 (17.2%)	115 (26.1%)	67 (15.2%)	105 (23.8%)
Posterior-superior alveolar nerve block	77 (17.5%)	88 (20.0%)	107 (24.3%)	73 (16.6%)	96 (21.8%)
Greater palatine nerve and lesser palatine nerves block	63 (14.3%)	68 (15.4%)	111 (25.2%)	79 (17.9%)	120 (27.2%)
Anterior-superior alveolar nerve block	57 (12.9%)	63 (14.3%)	110 (24.9%)	84 (19.0%)	127 (28.8%)
Maxillary nerve blocks	47 (10.7%)	62 (14.1%)	105 (23.8%)	81 (18.4%)	146 (33.1%)
Sphenopalatine nerve block	23 (5.2%)	42 (9.5%)	96 (21.8%)	97 (22.0%)	183 (41.5%)
Periodontal ligament technique	211 (47.8%)	105 (23.8%)	77 (17.5%)	23 (5.2%)	25 (5.7%)
Inferior alveolar nerve block (IANB)	179 (40.6%)	146 (33.1%)	81 (18.4%)	22 (5.0%)	13 (2.9%)
Intrapulpal injection technique	171 (38.8%)	101 (22.9%)	95 (21.5%)	28 (6.3%)	46 (10.4%)
Buccal nerve block	170 (38.5%)	94 (21.3%)	107 (24.3%)	36 (8.2%)	34 (7.7%)
Mental nerve block	133 (30.2%)	102 (23.1%)	91 (20.6%)	43 (9.8%)	72 (16.3%)
Gow-Gates technique	50 (11.3%)	70 (15.9%)	120 (27.2%)	82 (18.6%)	119 (27.0%)
Intraosseous injection technique	54 (12.2%)	71 (16.1%)	123 (27.9%)	64 (14.5%)	129 (29.3%)
Intraseptal injection technique	53 (12.0%)	69 (15.6%)	121 (27.4%)	69 (15.6%)	129 (29.3%)
Vazirani–Akinosi technique	33 (7.5%)	66 (15.0%)	119 (27.0%)	79 (17.9%)	144 (32.7%)

Data are presented as whole numbers and percentages.

**Table 5:** Experienced adverse effects following dental local anesthesia as reported by dental practitioners.

Characteristics		Frequency (n)	Percentage (%)
Experienced local complications following the administration of local anesthesia	No	70	15.9
	Yes	371	84.1
Types of local complications experienced following the application of local anesthesia (multiple-choice question)	Paresthesia	62	14.1
	Pain on injection site	228	51.7
	Sensory disorders	21	4.8
	Lack of effect	81	18.4
	Trismus	22	5.0
	Hematoma	68	15.4
	Ocular complications	42	9.5
	Other (infection, edema, etc.)	17	3.9
The specific type of local anesthetic associated with the occurrence of a local complication (multiple-choice question)	Articaine	295	66.9
	Lidocaine	50	11.3
	Mepivacaine	5	1.1
	Bupivacaine	2	0.5
	Prilocaine	0	0
	Topical anesthetics	0	0
	Do not remember	66	15.0
The local anesthesia techniques associated with the occurrence of a local complication (multiple-choice question)	Buccal Infiltration	136	30.8
	Intraligamentary technique	63	14.3
	Palatal Infiltration	41	9.3
	Inferior alveolar nerve bock (IANB)	181	41.0
	Mental and incisive blocks	25	5.7
	Gow-Gates technique	25	5.7
	Maxillary blocks	15	3.4
	Other	18	4.1
	Do not remember	40	9.1
Experienced systemic complications following the administration of local anesthesia	No	255	57.8
	Yes	186	42.2
Types of systemic complications experienced following the application of local anesthesia (multiple-choice question)	Psychomotor response (vasovagal syncope, panic attack, hyperventilation)	166	37.6
	Toxic reaction	8	1.8
	Allergic reactions and anaphylaxis	14	3.2
	Malignant hyperpyrexia, methemoglobinemia	0	0
	Vasoconstrictor reaction	25	5.7
The specific type of local anesthetic associated with the occurrence of a systemic complication (multiple-choice question)	Articaine	352	79.8
	Lidocaine	59	13.4
	Mepivacaine	7	1.6
	Bupivacaine	0	0
	Prilocaine	0	0
	Topical anesthetics	0	0
	Do not remember	0	0
The local anesthesia techniques associated with the occurrence of a systemic complication (multiple-choice question)	Buccal Infiltration	78	17.7
	Intraligamentary technique	9	2.0
	Palatal infiltration	16	3.6
	Maxillary blocks	6	1.4
	Inferior alveolar nerve block (IANB)	80	18.1
	Other	0	0
	Do not remember	41	9.3

Data are presented as whole numbers and percentages.

**Table 6:** Generalized linear model (GLM) analysis of the relationship between dentists’ sociodemographic characteristics in relation to knowledge of local anesthetics and self-confidence in using different dental anesthetic techniques.

Independent Variable	Categories	Knowledge Test		Self-Confidence Level	
		OR (95% CI)	*p*	OR (95% CI)	*p*
Gender	Male	Reference		Reference	
	Female	1.83 (1.13–2.98)	0.014	0.63 (0.41–0.99)	0.047
Age group (years)	≤30	Reference		Reference	
	31–40	0.31 (0.13–0.80)	0.015	1.42 (0.59–3.42)	0.437
	41–50	0.16 (0.05–0.55)	0.004	1.40 (0.44–4.46)	0.568
	≥51	0.29 (0.06–1.33)	0.112	0.77 (0.19–3.16)	0.715
Academic qualification	DMD	Reference		Reference	
	MSc	0.57 (0.19–1.67)	0.307	1.12 (0.42–3.01)	0.812
	PhD	2.12 (0.81–5.54)	0.126	1.72 (0.68–4.37)	0.247
Specializations in dental practice	General dentistry	Reference		Reference	
	Endodontic	2.12 (0.73–6.14)	0.166	1.23 (0.46–3.29)	0.678
	Oral surgery	7.04 (1.71–29.07)	0.007	4.91 (1.29–18.72)	0.020
	Oral medicine	0.14 (0.11–1.79)	0.131	1.324 × 10^−10^	1.000
	Pediatrics	0.91 (0.28–2.93)	0.876	0.52 (0.17–1.60)	0.256
	Orthodontics	1.38 (0.29–6.63)	0.688	0.16 (0.03–0.88)	0.035
	Periodontics	0.43 (0.11–1.59)	0.204	0.74 (0.23–2.32)	0.060
	Prosthodontics	0.45 (0.14–1.39)	0.165	0.82 (0.29–2.29)	0.704
	Family dentistry	0.28 (0.05–1.72)	0.170	0.20 (0.03–1.21)	0.079
Practice setting	Health center—primary care	Reference		Reference	
	Private practice—primary care	1.02 (0.60–1.73)	0.941	1.09 (0.68–1.76)	0.709
	Secondary and tertiary care	1.13 (0.39–3.22)	0.818	1.29 (0.53–3.14)	0.574
	Dental study	0.43 (0.15–1.25)	0.121	0.84 (0.29–2.49)	0.759
Number of patients per day (years)	1–5	Reference		Reference	
	6–10	0.35 (0.12–1.04)	0.058	1.36 (0.56–3.32)	0.506
	≥11	0.28 (0.09–0.83)	0.023	1.15 (0.46–2.83)	0.769
Working experience (years)	1–5	Reference		Reference	
	6–10	0.62 (0.24–1.63)	0.334	1.03 (0.41–2.56)	0.955
	11–20	1.07 (0.35–3.27)	0.901	0.88 (0.31–2.57)	0.826
	≤21	0.44 (0.11–1.78)	0.249	1.81 (0.48–6.79)	0.382
Experienced local adverse reactions	No	Reference		Reference	
	Yes	0.96 (0.52–1.76)	0.898	1.13 (0.66–1.96)	0.639
Experienced systemic adverse reactions	No	Reference		Reference	
	Yes	0.82 (0.53–1.29)	0.393	1.12 (0.75–1.68)	0.587

Reference knowledge or confidence level category is “low”. OR, odds ratio; 95% CI, 95% confidence interval.

## Data Availability

Data are available upon request from the corresponding author.
